# Physiological and Gene Expression Responses of Six Annual Ryegrass Cultivars to Cobalt, Lead, and Nickel Stresses

**DOI:** 10.3390/ijms222413583

**Published:** 2021-12-18

**Authors:** Siyu Qiao, Ye Tao, Qinghua Shan, Jingang Wang, Tuanyao Chai, Shufang Gong, Kun Qiao

**Affiliations:** 1 College of Horticulture and Landscape Architecture, Northeast Agricultural University, Harbin 150030, China; siyuqiao1997@163.com (S.Q.); ty18646408777@163.com (Y.T.); sqh19971001@163.com (Q.S.); wangjingang99@neau.edu.cn (J.W.); 2 College of Life Science, University of Chinese Academy of Sciences, Beijing, 100049, China; tychai@ucas.ac.cn

**Keywords:** ryegrass, Co/Pb/Ni tolerance, physiological indexes, gene expression

## Abstract

Heavy metals negatively affect soil quality and crop growth. In this study, we compared the tolerance of six ryegrass cultivars to cobalt (Co^2+^), lead (Pb^2+^), and nickel (Ni^2+^) stresses by analyzing their physiological indexes and transcript levels of genes encoding metal transporters. Compared with the other cultivars, the cultivar Lm1 showed higher germination rates and better growth under Co^2+^, Pb^2+^, or Ni^2+^ treatments. After 48 h of Co^2+^ treatment, the total antioxidant capacity of all six ryegrass cultivars was significantly increased, especially that of Lm1. In contrast, under Pb^2+^ stress, total antioxidant capacity of five cultivars was significantly decreased, but that of Lm1 was unaffected at 24 h. Staining with Evans blue dye showed that the roots of Lm1 were less injured than were roots of the other five ryegrass cultivars by Co^2+^, Pb^2+^, and Ni^2+^. Lm1 translocated and accumulated lesser Co^2+^, Pb^2+^, and Ni^2+^ than other cultivars. In Lm1, genes encoding heavy metal transporters were differentially expressed between the shoots and roots in response to Co^2+^, Pb^2+^, and Ni^2+^. The aim of these researches could help find potential resource for phytoremediation of heavy metal contamination soil. The identified genes related to resistance will be useful targets for molecular breeding.

## 1. Introduction

Heavy metals come from both the natural environment and human activities [1]. Heavy metal contaminants in soil are derived from traffic emissions (automobile exhaust, fuel combustion, tire wear particles), industrial emissions (mining of mineral resources, metal processing and smelting, coal combustion), and agricultural emissions (sewage irrigation and pesticide sprays) [2,3]. Common heavy metals are not naturally degraded and accumulate continuously in the environment. They pose a serious threat to human health and food security [4,5].

Some heavy metals such as cobalt (Co), nickel (Ni), manganese (Mn), zinc (Zn), copper (Cu), and iron (Fe) are essential micronutrients for plants. Cobalt plays an important role in plant growth and development. It is related not only to pigmentation in the leaves of legumes, but also the production of secondary metabolites, such as betaine [6]. However, excess Co in plants can disrupt many physiological, biochemical, and metabolic processes. Nickel is also an essential microelement for plant growth and development. It is a component of several enzymes (e.g., glyoxalase and urease) required for nitrogen metabolism, and plays an important role in nitrogen assimilation. Moreover, Ni can enhance the activity of various enzymes, and participate in resistance to abiotic stress. Therefore, Ni-deficient plants show symptoms, such as growth retardation and reduced nitrogen metabolism and iron absorption [7,8,9]. However, excess Ni can reduce the seed germination rate, negatively affect photosynthesis and respiration, and cause yellowing and necrosis of the leaves [10].

Lead (Pb) is a non-essential element in plants. It can inhibit seed germination and plant growth. Excess Pb can directly inhibit leaf development and the elongation of stems and roots [11]. It also affects glucose metabolism and stimulates the oxidation of indole-3 acetic acid. Consequently, Pb negatively affects plant growth, biomass, and cell growth [12].

Annual ryegrass (*Lolium multiflorum* Lam.) is a widely distributed good-quality forage grass, and it can be used as an indicator plant in heavy metal-contaminated environments [13]. Compared with white clover and alfalfa, annual ryegrass is more tolerant to Cd [14]. Compared with Cd-sensitive cultivars, the more Cd-tolerant ryegrass cultivars accumulate and transport less Cd [15]. However, little is known about the effects of other heavy metals, including Co^2+^, Pb^2+^, and Ni^2+^, on gene expression and the growth and development of annual ryegrass cultivars.

In this study, we analyzed physiological parameters of six ryegrass cultivars treated with Co^2+^, Pb^2+^, or Ni^2+^, and determined changes in the transcript levels of genes encoding heavy metal transporters. As a preliminary analysis of the effects of these heavy metals on ryegrass, we determined the relative inhibition rate of germination, relative inhibition rate of shoot length, and relative inhibition rate of root length of six ryegrass cultivars treated with solutions of Co^2+^, Pb^2+^, or Ni^2+^. The effects of these heavy metals on root cell survival were determined using Evan’s blue staining. We determined the total antioxidant capacity of the six cultivars under Co^2+^, Pb^2+^, or Ni^2+^ stress. To analyze the uptake and translocation of heavy metals, we conducted milestone microwave digester and inductively coupled plasma mass spectrometry (ICP-MS) analyses to determine the contents of heavy metals in roots and shoots. The effects of Co^2+^, Pb^2+^, and Ni^2+^ treatments on the transcript levels of genes encoding heavy metal transporters were determined by RT-qPCR. The results of this study will be useful for identifying hyperaccumulator ryegrass varieties to phytoremediate soils contaminated with heavy metals. In addition, genes related to resistance will be useful targets for selective and molecular breeding strategies.

## 2. Results

### 2.1. Effects of Co^2+^, Pb^2+^, and Ni^2+^ on Growth of Six Ryegrass Cultivars

**Figure 1:**
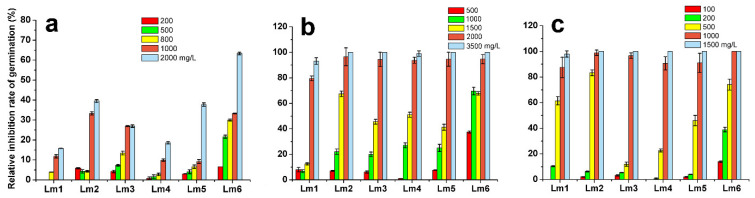
Relative inhibition rate of germination of six ryegrass cultivars under different concentrations of Co^2+^, Pb^2+^, and Ni^2+^. The ryegrass seeds were germinated for 5 d on sterile filter paper containing different concentrations of Co (**a**), Pb (**b**), and Ni (**c**). CoCl_2_ concentrations: 0, 200, 500, 800, 1000, 2000 mg/L; Pb(NO_3_)_2_ concentrations: 0, 500, 1000, 1500, 2000, and 3500 mg/L; NiSO_4_ concentrations: 0, 100, 200, 500, 1000, and 1500 mg/L. The plates were divided into four zones, each with 25 seeds.

As the Co^2+^ concentration increased, the germination of the six ryegrass cultivars gradually decreased (Figure 1). As shown in Figure 1a and Figure S1, the germination of Lm2, Lm3, Lm4, Lm5, and Lm6 were inhibited by the 200–500 mg/L CoCl_2_ treatments, but that of Lm1 was unaffected. In the 2000 mg/L CoCl_2_ treatment, the relative inhibition rate of germination of Lm1 was 10%, which was significantly lower than those of the other ryegrass cultivars.

The Pb treatments significantly inhibited the germination of all six ryegrass cultivars (Figure 1b, Figure S2). Notably, the relative inhibition rate of germination was lower in Lm4 than in the other cultivars in the 500 mg/L Pb treatment, and the relative inhibition rate of germination of Lm1 was significantly lower than that of other cultivars in the 1000–1500 mg/L Pb treatments. In the 3500 mg/L Pb treatment, seed germination was completely inhibited in all cultivars.

The relative inhibition rate of germination was lower for Lm1 than for the other cultivars in the treatments with lower concentrations of Ni (Figure 1c, Figure S3). However, as the Ni concentration increased, the relative inhibition rate of germination of Lm1 enhanced, and was not significantly different from those of the other cultivars in the 200–1500 mg/L Ni treatments.

### 2.2. Effects of Co^2+^, Pb^2+^, Ni^2+^ on Physiological Indexes of Six Ryegrass Cultivars

**Figure 2:**
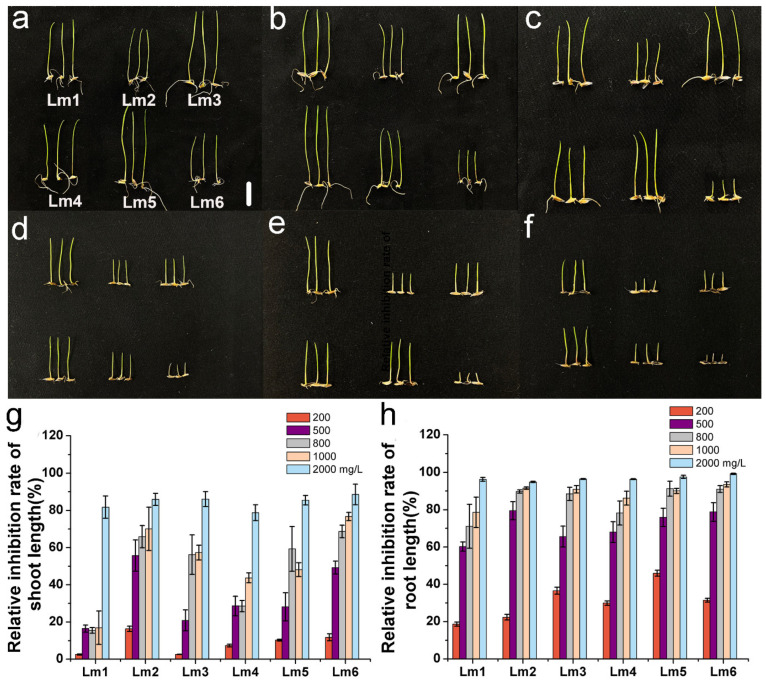
Phenotypic analysis of six ryegrass cultivars treated with different Co^2+^ concentrations. All seeds were germinated with 0 (**a**), 200 (**b**), 500 (**c**), 800 (**d**), 1000 (**e**), and 2000 mg/L (**f**) CoCl_2_. Growth was observed and photographed, and relative inhibition rate of shoot length (**g**), and relative inhibition rate of root length (**h**) were analyzed at 6 d. Scale bars: 10 mm.

To understand the variations in heavy metal tolerance among the six ryegrass cultivars, we determined the relative inhibition rates of shoot length and root length of each cultivar under Co^2+^, Pb^2+^, or Ni^2+^ stress. The relative inhibition rate of shoot length of Lm1 was significantly lower than those of the other cultivars in the 200 to 1000 mg/L CoCl_2_ treatments (Figure 2a–g). The relative inhibition rate of root length was also slightly weaker in Lm1 than in the other cultivars in the 200 to 1000 mg/L CoCl_2_ treatments. However, root growth was almost completely inhibited in the 2000 mg/L CoCl_2_ treatment (Figure 2h).

**Figure 3:**
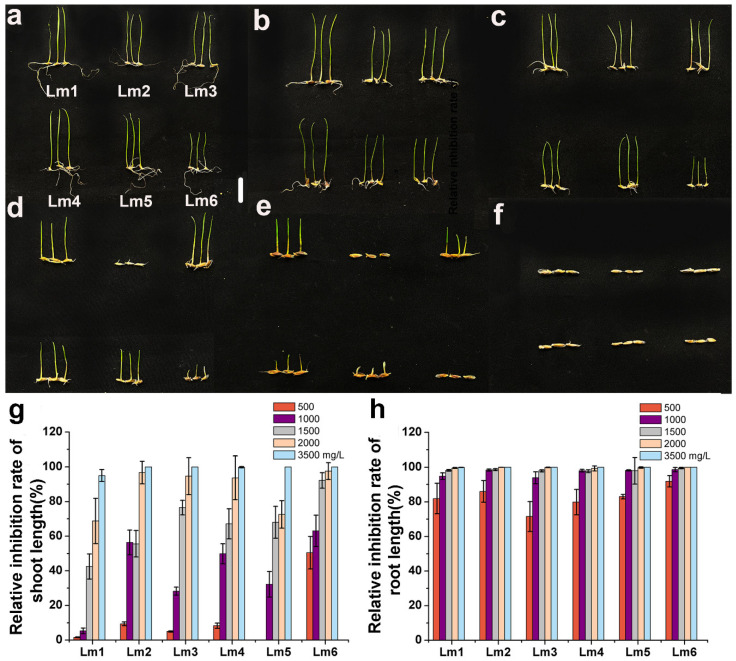
Phenotypic analysis of six ryegrass cultivars treated with Pb(NO_3_)_2_ at 0 (**a**), 500 (**b**), 1000 (**c**), 1500 (**d**), 2000 (**e**), and 3500 (**f**) mg/L. All seeds were germinated with different concentrations of Pb(NO_3_)_2_. Growth was observed and photographed (**a**–**f**), and relative inhibition rate of shoot length (**g**), and relative inhibition rate of root length (**h**) were analyzed at 6 d. Scale bars: 10 mm.

In the Pb treatments, the relative inhibition rate of shoot length was weaker in Lm1 than in the other cultivars in the 1000 to 1500 mg/L Pb treatments (Figure 3a–g). The root growth of cultivars was all inhibited by Pb stress, and the relative inhibition rates of root length was not significantly different among the six ryegrass cultivars (Figure 3h). The seed germination of Lm5 was completely inhibited (Figure 3f).

**Figure 4:**
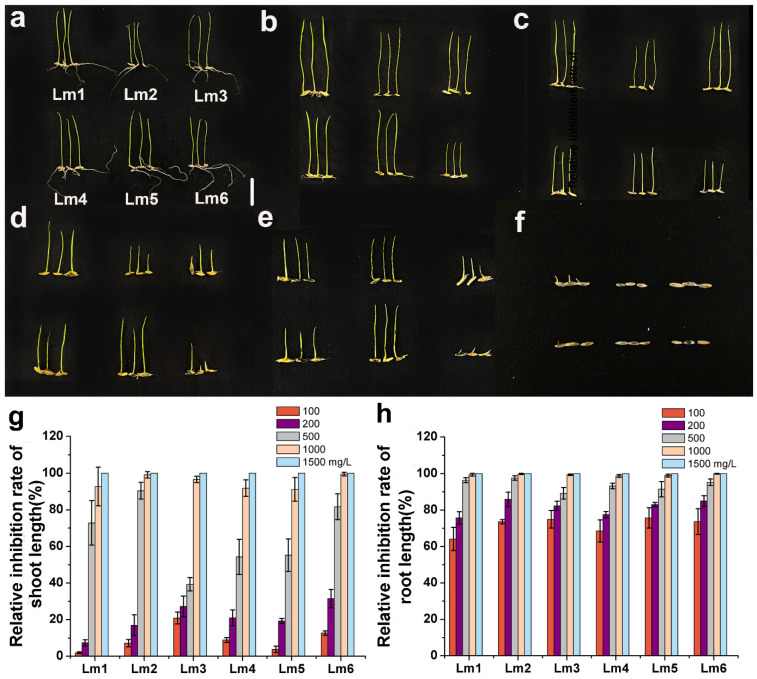
Phenotypic analysis of six ryegrass cultivars treated with NiSO_4_ at 0 (**a**), 100 (**b**), 200 (**c**), 500 (**d**), 1000 (**e**), and 1500 (**f**) mg/L. All seeds were germinated with different concentrations of NiSO_4_. Growth was observed and photographed (**a**–**f**), and relative inhibition rate of shoot length (**g**), and relative inhibition rate of root length (**h**) were analyzed at 6 d. Scale bars: 10 mm.

The relative inhibition rate of shoot length of Lm1 was lower than those of the other five cultivars in the 100 and 200 mg/L NiSO_4_ treatments, but significantly higher than those of Lm3, Lm4, and Lm5 in the 500 mg/L Ni treatment (Figure 4a–g). The relative inhibition rates of root length of Lm1 were not significantly different from those of the other five ryegrass cultivars in the Ni treatments (Figure 4h).

### 2.3. Effects of Heavy Metals on the Total Antioxidant Capacity of Six Ryegrass Cultivars

**Figure 5:**
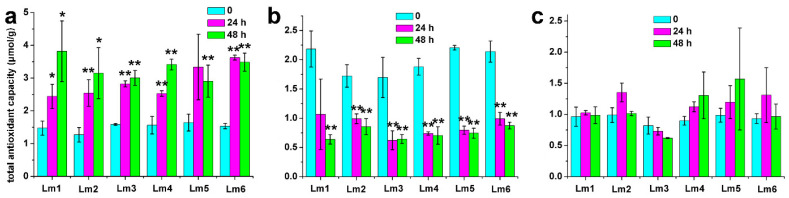
Total antioxidant capacity of six ryegrass cultivars treated with Co^2+^, Pb^2+^, or Ni^2+^. (**a**) total antioxidant capacity of six ryegrass cultivars treated with 500 mg/L CoCl_2_; (**b**) total antioxidant capacity of six ryegrass cultivars treated with 1000 mg/L Pb(NO_3_)_2_; (**c**) total antioxidant capacity of six ryegrass cultivars treated with 500 mg/L NiSO_4_. Significant differences were determined by *t*-test in Excel 2010 (** *p* < 0.01, * 0.01 < *p* < 0.05).

The antioxidant system allows plants to remove toxic reactive oxygen species (ROS), and is often used as an indicator of plant stress resistance. The total antioxidant capacity of six ryegrass cultivars differed between the control and the heavy metal treatments. The total antioxidant capacity of the six cultivars was increased in response to Co treatments (Figure 5a), decreased in response to Pb treatments (Figure 5b), and unchanged by Ni treatments (Figure 5c). These findings indicated that the ryegrass cultivars might be tolerant to Co but sensitive to Pb. The concentrations of Ni used in this experiment did not affect the total antioxidant capacity, so could not distinguish the cultivars in terms of their Ni tolerance (Figure 5).

### 2.4. Toxicity Symptoms in Roots of Six Ryegrass Cultivars Treated with Heavy Metals

**Figure 6:**
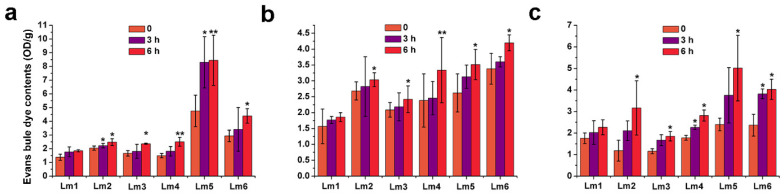
Evans blue staining analysis of roots of six ryegrass cultivars subjected to Co^2+^ (**a**), Pb^2+^ (**b**), or Ni^2+^ (**c**) stress for 3 and 6 h. Roots of treated samples were washed with distilled water, and soaked in 0.025% Evan’s blue dye for 15 min. Stained samples were cleared with distilled water, and then observed and photographed under a BX41 light microscope. Significant differences were determined by *t*-test in Excel 2010 (** *p* < 0.01, * 0.01 < *p* < 0.05).

At high concentrations, heavy metals can damage plant tissues, especially roots. To observe damage caused by heavy metals, the roots of the six ryegrass varieties were soaked in solutions containing Co^2+^, Pb^2+^, or Ni^2+^, and then stained with Evan’s blue dye, which stains dead cells blue. In Lm2, Lm3, Lm4, Lm5, and Lm6, the roots treated with Co^2+^, Pb^2+^, or Ni^2+^ showed significantly stronger Evan’s blue contents than did the untreated roots (*p* < 0.05). In Lm1, however, the Evan’s blue contents in roots treated with heavy metals for 3 and 6 h were not significantly different from that in the control (Figure 6a–c). The staining of all cultivars’ root cells was not significantly different between the 3 h and 6 h time points, with or without heavy metal treatments (Figures S4–S6).

### 2.5. Comprehensive Evaluation of Co^2+^, Pb^2+^, and Ni^2+^ Tolerance of Six Ryegrass Cultivars

Plants’ tolerance to heavy metals is complex. Therefore, it is poorly represented using a single index of tolerance. In this study, the effects of different concentrations of Co^2+^, Pb^2+^, and Ni^2+^ on several morphological and physiological indexes of six ryegrass cultivars were studied, and tolerance was comprehensively evaluated using the membership function method [16,17]. The six cultivars were ranked, from most to least tolerant, as follows: to Co^2+^, Lm1 > Lm6 >Lm4> Lm3 > Lm5 > Lm2 (Table S2); to Pb^2+^, Lm1 > Lm5 > Lm2 > Lm6 > Lm4 > Lm3 (Table S3); and to Ni^2+^, Lm1 > Lm3 > Lm4 > Lm2 > Lm5 > Lm6 (Table S4). Thus, compared with the other five cultivars, Lm1 showed stronger tolerance to Co^2+^, Pb^2+^, and Ni^2+^.

### 2.6. Heavy Metal Accumulation in Shoots and Roots of Six Ryegrass Cultivars

**Figure 7:**
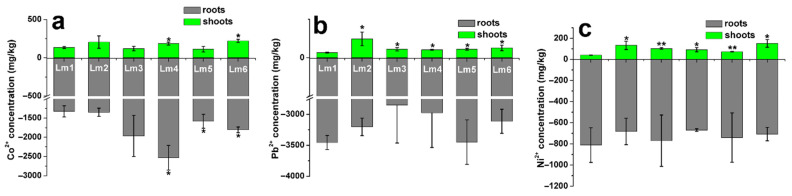
Concentrations of Co^2+^, Pb^2+^, and Ni^2+^ in shoots and roots of six ryegrass cultivars subjected to Co^2+^, Pb^2+^, or Ni^2+^ stress for 24 h. Milestone microwave digester and ICP-MS was used to digest and detect heavy metals in shoots and roots. Data are mean ± standard error from three independent experiments. Asterisks indicate significant differences from control levels (** *p* < 0.01, * 0.01 < *p* < 0.05). (**a**) Co^2+^ concentrations in shoots and roots of six ryegrass cultivars under CoCl_2_ treatment; (**b**) Pb^2+^ concentrations in shoots and roots of six ryegrass cultivars under Pb(NO_3_)_2_ treatment; (**c**) Ni^2+^ concentrations in shoots and roots of six ryegrass cultivars under NiSO_4_ treatment.

To understand the translocation and accumulation of Co^2+^, Pb^2+^, and Ni^2+^ in the six ryegrass cultivars, we conducted milestone microwave digester and ICP-MS analyses. In the Co^2+^ treatment, the Co^2+^ concentration in shoots was lower in Lm1 than in Lm4 and Lm6 (*p* < 0.05), but was not significantly different between Lm1 and the other three cultivars (Figure 7a). The Pb^2+^ and Ni^2+^ concentrations in shoots were significantly lower in Lm1 than in the other five ryegrass cultivars (*p* < 0.05, Figure 7b,c). These results showed that Lm1 translocated a small amount of heavy metals from roots to shoots under Co^2+^, Pb^2+^, and Ni^2+^ stresses.

### 2.7. Changes in Transcript Levels of Genes Encoding Heavy Metal Transporters in Response to Heavy Metal Treatments

**Figure 8:**
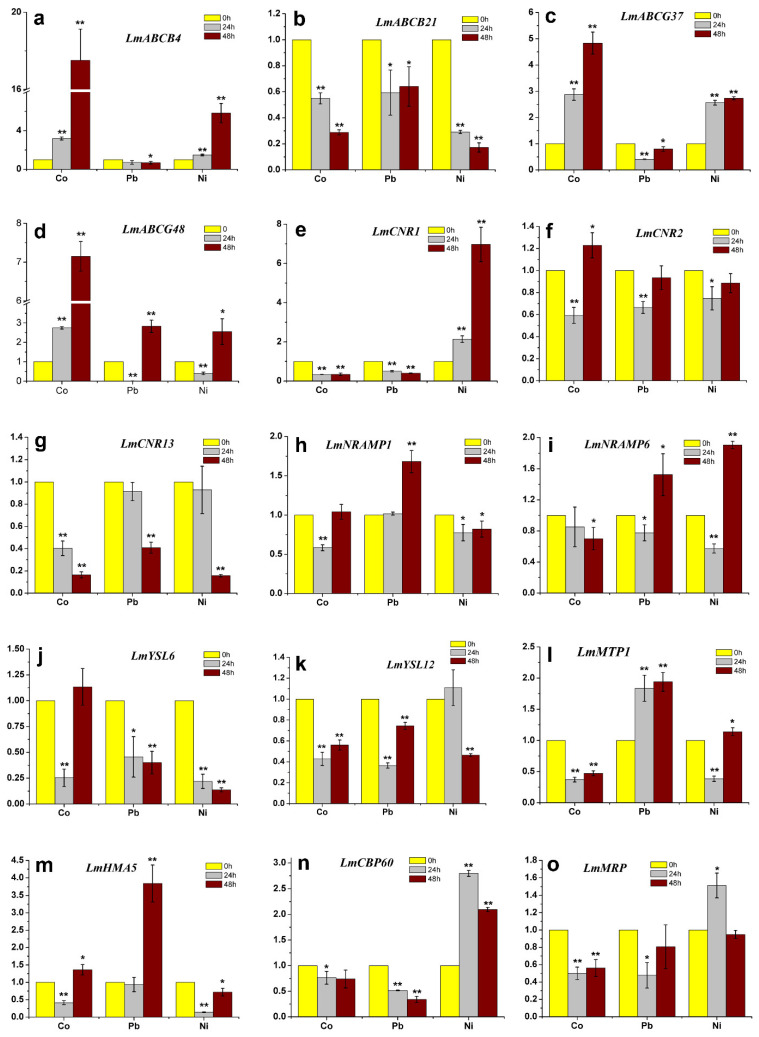
Relative transcript levels of 15 genes encoding transporters in Lm1 shoots. Gene transcript levels were determined by RT-qPCR at 24 and 48 h of Co^2+^, Pb^2+^, or Ni^2+^ treatment. (**a**) *LmABCB4*, (**b**) *LmABCB21*, (**c**) *LmABCG37*, (**d**) *LmABCG48*, (**e**) *LmCNR1*, (**f**) *LmCNR2*, (**g**) *LmCNR13*, (**h**) *LmNRAMP1*, (**i**) *LmNRAMP6*, (**j**) *LmYSL6*, (**k**) *LmYSL12*, (**l**) *LmMTP1*, (**m**) *LmHMA5*, (**n**) *LmCBP60*, (**o**) *LmMRP*. Data are means ± SE of three independent samples. Data were analyzed by *t*-test, and significant differences are indicated by asterisk (* 0.01 < *p* < 0.05, ** *p* < 0.01).

**Figure 9:**
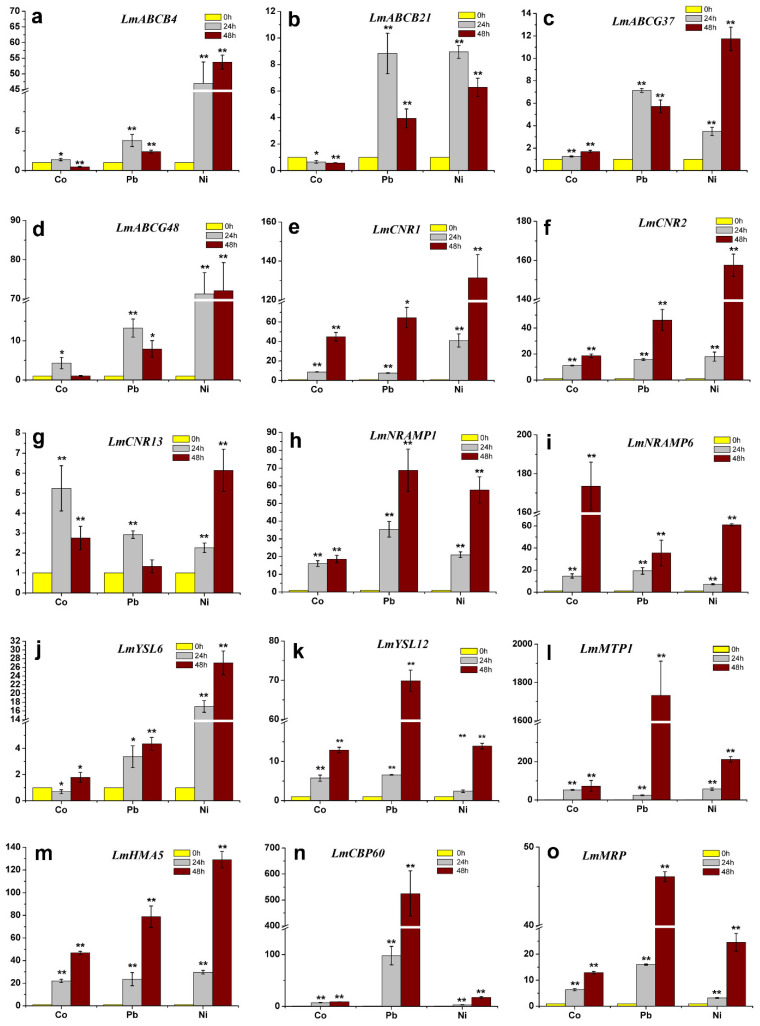
Relative transcript levels of 15 genes encoding transporters in Lm1 roots. Gene transcript levels were determined by RT-qPCR at 24 and 48 h of Co^2+^, Pb^2+^, or Ni^2+^ treatment. (**a**) *LmABCB4*, (**b**) *LmABCB21*, (**c**) *LmABCG37*, (**d**) *LmABCG48*, (**e**) *LmCNR1*, (**f**) *LmCNR2*, (**g**) *LmCNR13*, (**h**) *LmNRAMP1*, (**i**) *LmNRAMP6*, (**j**) *LmYSL6*, (**k**) *LmYSL12*, (**l**) *LmMTP1*, (**m**) *LmHMA5*, (**n**) *LmCBP60*, (**o**) *LmMRP*. Data are means ± SE of three independent samples. Data were analyzed by *t*-test, and significant differences are indicated by asterisk (* 0.01 < *p* < 0.05, ** *p* < 0.01).

The comprehensive evaluation showed that, compared with the other five cultivars, Lm1 had better tolerance to Co^2+^, Pb^2+^, and Ni^2+^. To explore the mechanisms of its tolerance, we conducted RT-qPCR analyses to determine the transcript levels of 15 genes encoding heavy metal transporters in the shoots and roots of Lm1 plants treated with Co^2+^, Pb^2+^ and Ni^2+^. As shown in Figure 8 and Figure 9 in the 500 mg/L Co^2+^ treatment, the transcript levels of *ABCB4*, *ABCG37*, and *ABCG48* in Lm1 shoots increased from 24 to 48 h (Figure 8a,c,d; *p* < 0.05), and those of *CNR2* and *HMA5* slightly increased (Figure 8f,m; *p* < 0.05). In contrast, the transcript levels of *ABCB21*, *CNR1*, *CNR13*, *NRAMP1*, *NRAMP6*, *YSL6*, *YSL12*, *MTP1*, *CBP60*, and *MRP* decreased (Figure 8b,e,g–l,n,o).

The transcript levels of *ABCB4*, *ABCB21*, ABCG37, *CNR1*, *CNR2*, *CNR13*, *YSL6*, *YSL12*, *CBP60*, and *MRP* in Lm1 shoots were decreased at 24 and 48 h of the 1000 mg/L Pb^2^^+^ treatment (Figure 8a–c,e,f,g,i–k,n,o), but those of *ABCG48*, *NRAMP1*, *NRAMP6*, *MTP1*, and *HMA5* were increased, compared with their respective levels at 0 h (Figure 8d,h,i,l,m; *p* < 0.05). In the shoots of Lm1, the transcript levels of *ABCB4*, *ABCG37*, *ABCG48*, *CNR1*, *NRAMP6*, *MTP1*, *CBP60*, and *MRP* increased in the 500 mg/L Ni^2+^ treatment (Figure 8a,c–e,i,n,o; *p* < 0.05), while those of *ABCB21*, *CNR2*, *CNR13*, *NRAMP1*, *YSL6***,** *YSL12*, and *HMA5* decreased (Figure 8b,f–h,j,k,m).

The transcript levels of *ABCB4*, *ABCG37*, *ABCG48*, *CNR1*, *CNR2*, *CNR13*, *NRAMP1*, *NRAMP6*, *YSL6*, *YSL12*, *MTP1*, *HMA5 CBP60*, and *MRP* in roots of Lm1 were markedly increased in the 500 mg/L Co^2+^ treatment (Figure 9c–i,k–o; *p* < 0.05), while those of *ABCB4* and *YSL6* were slightly higher than in the control (Figure 9a,j; *p* < 0.05). In the roots, only *ABCB21* showed lower transcript levels in the 500 mg/L Co^2+^ treatment than in the control (Figure 9b).

In the 1000 mg/L Pb^2^^+^ and 500 mg/L Ni^2+^ treatments, 11 genes were up-regulated in the roots, compared with their respective levels in the control (Figure 9; *p* < 0.05).

## 3. Discussion

### 3.1. Effects of Heavy Metals on Seed Germination of Six Ryegrass Cultivars

Seed germination is the first important stage of the plant life cycle, and the germination rate reflects seedling emergence. Seed germination is a prerequisite for plant survival under stress conditions. Previous studies have reported decreased rates of seed germination of alfalfa (*Medicago sativa* L.) and rice under Co stress [18], shepherd’s purse and black bean under Pb stress [19,20], and millet and oat under Ni stress [7]. In the present study, the seed germination of six ryegrass cultivars was inhibited by high concentrations of Co^2+^, Pb^2+^, and Ni^2+^. However, the relative inhibition rate of germination of Lm1 was lower than those of the other five ryegrass cultivars under Co^2+^, Pb^2+^ and Ni^2+^ stresses (Figure 1). Thus, we concluded that Lm1 had better tolerance to Co, Pb, and Ni stresses, compared with the other five ryegrass cultivars.

### 3.2. Effects of Co^2+^, Pb^2+^, and Ni^2+^ Stresses on Ryegrass Growth

Plant height, root length, and biomass are the most basic growth indexes, and they are important criteria to evaluate the stress tolerance of plants and the degree of stress. Previous studies have shown that the accumulation of heavy metals affects plant growth and development, and that seedlings exposed to heavy metals become more sensitive to external environmental stimuli [14,15]. In another study, the shoot length and root length of rice seedlings tended to decrease as Co^2+^ stress became more severe [18]. In *Chenopodium murale* treated with 300, 400, and 500 mg/kg Pb^2+^, the shoot length, root length, and biomass decreased with increasing Pb^2+^ concentrations [21]. In rice, the shoot length, root length, dry weight, and fresh weight of plants under 200 μM Ni^2+^ stress were 31.05%, 32.07%, 65.09%, and 63.88% lower, respectively, than in the control, and Ni^2+^ stress had a stronger inhibitory effect on biomass than on plant height [22]. In the present study, the relative inhibition rates of shoot length and root length were lower in Lm1 than in the other cultivars under Co^2+^, Pb^2+^ and Ni^2+^ stresses, indicating that it was more tolerant to heavy metal stresses. We observed that all ryegrass cultivars showed inhibited growth in the heavy metal treatments, compared with the control, and that heavy metals had a stronger inhibitory effect on root length than on shoot length.

### 3.3. Effects of Co^2+^, Pb^2+^, and Ni^2+^ Stresses on Total Antioxidant Capacity of Ryegrass

Plants produce ROS under heavy metal stress. To maintain the balance of ROS in vivo, plants have evolved a complex antioxidant system to protect their cellular structure [23]. The activities of antioxidant enzymes (such as superoxide dismutase, SOD; catalase, CAT; and ascorbate peroxidase, APX) often increase under stress conditions. Superoxide dismutase decomposes ROS into H_2_O_2_, which is then removed by CAT and APX [23]. Studies on rice plants subjected to 100 μM Pb^2+^ stress showed that SOD, APX, and glutathione reductase activities increased, and the levels of antioxidant substances, such as ascorbic acid and glutathione decreased [24]. In *Camelina sativa,* the activities of SOD, CAT, and APX were up-regulated under Ni^2+^ treatment [25]. In contrast, in *Brassica juncea* subjected to a high Cu^2+^ treatment, SOD, CAT, and peroxidase activities decreased [19]. In this study, the total antioxidant capacity of six ryegrass cultivars was significantly increased by a 500 mg/L Co^2+^ treatment, unaffected by a 500 mg/L Ni^2+^ treatment, and significantly decreased by a 1000 mg/L Pb^2+^ treatment. The very high concentration of Pb^2+^ may have damaged plant cells or down-regulated other non-enzymatic antioxidants (such as ascorbic acid), leading to decreased total antioxidant capacity [26].

### 3.4. Accumulation and Translocation of Heavy Metals in Ryegrass Plants

The Co^2+^, Pb^2+^, and Ni^2+^ concentrations in shoots were generally lower in Lm1 than in the other ryegrass cultivars, indicating that Lm1 accumulated less heavy metals than did the other cultivars. Because it accumulated fewer heavy metals, it was able to tolerate higher concentrations of Co^2+^, Pb^2+^, Ni^2+^. This explains why Lm1 showed stronger tolerance than that of other ryegrass cultivars to heavy metals.

Plants have different types of transporters that help to translocate heavy metal ions and maintain the ion balance in the cytoplasm. We detected increased transcript levels of some genes encoding transporters in Lm1 treated with Co^2+^, Pb^2+^, or Ni^2+^. A superfamily of ATP-binding cassette (ABC) transporters (including ABCA-ABCH types) is involved in heavy metal transport. For example, in hybrid poplar, PcABCG36 localized on the plasma membrane transports Pb^2+^ from the cytoplasm across the plasma membrane [27]. In rice, OsABCG36 inhibits Cd accumulation in root cells and enhances Cd tolerance [28]. In *Arabidopsis*, AtABCG37 is located in the plasma membrane of root cells, and it regulates auxin translocation [29]. A previous study reported that *LmABCG37* in ryegrass roots was up-regulated by Cd treatment [15], and also by treatments with Co, Pb, or Ni, suggesting that it is involved in the translocation of heavy metal ions in roots. In *Arabidopsis*, AtABCB21 is located in the plasma membrane of root pericycle cells, where it imports or exports auxin, whereas AtABCB4 is responsible for exporting auxin [30]. In the present study, *LmABCB21* was down-regulated in Lm1 shoots under Co^2+^, Pb^2+^ and Ni^2+^ stresses, indicating that LmABCB21 might not be involved in heavy metal accumulation and/or transport from roots to shoots. In contrast, *LmABCG48* was up-regulated in shoots and roots of Lm1 plants treated with Co^2+^, Pb^2+^, or Ni^2+^. Therefore, LmABCG48 might be involved in the efflux of excess heavy metals from the plasma membrane. In addition, *LmABCG4* and *LmABCG37* had higher expression at Co^2+^ and Ni^2+^ treatments in Lm1 shoots and roots, implying that they both were involved with Co^2+^ and Ni^2+^ translocation from roots to shoots.

A previous study found that the rice *OsHMA5* mutant accumulated lower levels of copper ions in the xylem sap, suggesting that OsHMA5 may mediate the translocation of heavy metals from the roots to the shoots through the vascular bundle [31,32]. A homolog of HMA5 in poplar, PtHMA4, was also found to be localized at the plasma membrane. Its encoding gene, *PtHMA4,* was up-regulated in the roots of plants treated with Co or Pb, suggesting that it may be involved in the transport of Co and Pb from roots to shoots [33]. We detected increased transcript levels of *LmHMA5* in shoots and roots of Lm1 treated with Pb, suggesting that LmHMA5 might be involved in the translocation of Pb^2+^ from the roots to the shoots.

The NRAMP proteins are membrane-located transporters of heavy metals including Co^2+^, Pb^2+^, and Ni^2+^. In *Chlamydomonas*, CrNRAMP1 is localized at the plasma membrane, and the CrNRAMP1 mutant showed increased Co tolerance [34]. In poplar, PtNARMP1.3 located in the plasma membrane transports Pb^2+^ from outside the cells to the cytoplasm for chelation [35]. In *Thlaspi japonicum*, TjNARMP4 is located on the vacuolar membrane, and is involved in Ni^2+^ accumulation and translocation. Overexpression of *TjNARMP4* increased the contents of Ni in cells and enhanced sensitivity to Ni^2+^ [36]. Two homologs of *NARMP4* in potato, *StNRAMP2* and *StNRAMP3*, were found to be significantly up-regulated in roots by a Ni^2+^ treatment, and were found to be involved in the absorption and translocation of Ni^2+^ [37]. In the present study, in Lm1 treated with heavy metals, the transcript levels of *LmNRAMP1* and *LmNRAMP6* were slightly increased in the shoots, but strongly increased in the roots. These results suggest that the proteins encoded by *LmNRAMP1* and *LmNRAMP6* participate in translocation of Co^2+^, Pb^2+^, and Ni^2+^, analogous to the roles of StNRAMP2 and StNRAMP3 in potato.

Metal tolerance proteins in the cation diffusion facilitator family are responsible for transporting heavy metal ions such as Co^2+^, Ni^2+^, and Cd^2+^. In *Thlaspi goesingense*, TgMTP1 is localized at the vacuolar membrane in shoots and roots, and it transports Ni^2+^ into the vacuole for chelation [38]. In wheat, TuMTP1 located on the vacuolar membrane transports excess Co^2+^ into vacuoles [39]. In the present study, the *LmMTP1* transcript levels were significantly higher in roots than in shoots. The probable role of its encoded protein is to transport Co^2+^, Pb^2+^, and Ni^2+^ into the vacuole of root cells for sequestration, because even small amounts of heavy metals can damage the shoots.

The YSL protein is mainly involved in Fe^2+^ and Mn^2+^ transport in cells, and is also related to Pb^2+^ and Ni^2+^ transport. In *Arabidopsis*, AtYSL4 is located on the vacuolar membrane and is responsible for transporting excess Ni^2+^ from cells out of vacuoles to relieve heavy metal toxicity [40]. The expression of *YSL7* in roots of *Brassica juncea* was found to be up-regulated under high Pb stress. Similarly, under Ni treatment, the transcript level of *BjYSL7* in the stem was found to be significantly increased. The overexpression of *BjYSL7* in tobacco significantly increased the Ni content in shoots, providing evidence that YSL7 is involved in transporting Ni from roots to shoots [41]. In Lm1, *LmYSL6* and *LmYSL12* transcript levels were higher in roots than in shoots, but they decreased under Co, Pb, and Ni treatments. Thus, some YSL proteins might be involved in heavy metal uptake into the roots, but not in their translocation to shoots.

The cell number regulator (CNR) proteins belong to the placenta-specific 8 family, and their role is to regulate cell number and fruit size. However, TaCNR2, TaCNR5, and TuCNR10 are involved in the accumulation and translocation of Cd^2+^, Zn^2+^, and Mn^2+^ in the roots of wheat [42,43]. In this study, we detected higher transcript levels of *LmCNR1*, *LmCNR2*, and *LmCNR13* in the roots of Lm1 treated with heavy metals, but they were down-regulated in shoots, implying that they do not participate in Co^2+^, Pb^2+^, and Ni^2+^ translocation.

CBP60 can participate in regulating salicylic acid biosynthesis, and *CBP60* in transgenic Arabidopsis is sensitive to abscisic acid and enhances the tolerance to drought stress [44]. Because NtCBP4 can reduce Ni^2+^ concentration and enhance Pb^2+^ accumulation, respectively. Therefore, tobacco NtCBP4 presents improved Ni^2+^ tolerance and Pb^2+^ sensitivity [45]. *LmCBP60* transcript levels in roots were significantly induced at Co^2+^, Pb^2+^, and Ni^2+^, and the expression level had 500 times than in the control under Pb^2+^ treatment.

MRP belongs to ABC transporter sequence of C subfamily, which may be associated with the translocation of Cd chelates or glutaminesynthease-Cd complexes in the vacuolar membrane. In addition, transcriptional levels of four *MRPs* are increased in treated roots from Arabidopsis [46]. *AtMRP3* transporter and its promoter increased their expression in treated with Cd, Ni, Co, Pb, and As in transgenic Arabidopsis and Nicotiana [47]. Moreover, *LmMRP*, such as *AtMRP3*, also had higher expression at Co^2+^, Pb^2+^, and Ni^2+^ in ryegrass roots.

In summary, the main findings in this study were that we screened out a ryegrass cultivar (Lm1) with strong tolerance to Co, Pb, and Ni through physiological and phenotypic analysis. However, the transcript levels of most genes (LmABCB4, LmABCG37, LmABCG48, LmCNR1, LmCNR2, LmCNR13, LmNARMP1, LmNARMP6, LmYSL6, LmYSL12, LmMTP1, LmHMA5, LmCBP60, and LmMRP) encoding heavy metal transporters were significantly higher in the heavy metal treatments than in the control in the roots. Moreover, in the shoots, only LmABCB4 and LmABCG37 were up-regulated in the Co and Ni treatments, and only LmABCG48 was up-regulated in all the heavy metal treatments (Figure 8 and Figure 9). These indicated that LmABCB4, LmABCG37, and LmABCG48 are key component of the response to heavy metal accumulation and translocation. Further research is required to explore their role in more detail. Most transporters are not expressed in the shoots of Lm1, then heavy metals may not be transported to the shoots through the xylem. This may explain why Lm1 showed higher tolerance to heavy metals, compared with the other five cultivars. A recent study found that intercropping of ryegrass and Indian mustard can improve phytoremediation of antibiotics [48]. The inspiration for us is to use ryegrass intercropping with other herbaceous plants to enhance stress resistance and reduce the content of heavy metals in soil.

## 4. Materials and Methods

### 4.1. Plant Materials and Tolerance Analysis

The seeds of six ryegrass cultivars (designated as Lm1, Lm2, Lm3, Lm4, Lm5, and Lm6) were purchased from grass industry groups (Table S1). The seeds were disinfested with 0.5% *v*/*v* NaClO_3_ for 15 min, washed five to six times with sterile distilled water, and then dried with filter paper. The disinfested seeds were placed evenly in Petri dishes containing a double layer of filter paper, and 7 mL treatment solution (CoCl_2_: 0, 200, 500, 800, 1000, 2000 mg/L; Pb(NO_3_)_2_: 0, 500, 1000, 1500, 2000, 3500 mg/L; NiSO_4_: 0, 100, 200, 500, 1000, 1500 mg/L) was added to each dish. Each treatment had four replicates with 25 seeds per group (100 seeds/treatment). During the experiment, the cultivation conditions were as follows: 12 h light/12 h dark photoperiod, 22 °C, and relative humidity of 60%. The treatment solution was added to each Petri dish each day, and the number of germinated seeds was recorded every day for 5 days. On day 6, the resulting seedings were put onto the 1/2 Murashige and Skoog solid media (Qingdao Hope Bio-technology Co. Ltd., Qingdao, China) and photographed. The shoot length and root length were detected by a vernier calipers.

### 4.2. Determination of Total Antioxidant Capacity

We used 14-day-old hydroponically grown seedlings in these experiments. Six cultivars of seedlings with similar growth were treated with 500 mg/L CoCl_2_, 1000 mg/L Pb(NO_3_)_2_, or 500 mg/L NiSO_4_ for 0 h, 24 h, and 48 h. The same heavy metal concentrations were used in the further experiments. Then, the total antioxidant capacity of ryegrass cultivars was performed using an extraction kit (Solarbio, Beijing, China). 0.1 g fresh treated ryegrass samples (the whole seedling) were collected and ground on ice with 1.0 mL pre-cooled extraction buffer in with a pestle and mortar. The mixture was then centrifuged at 10000 rpm/min at 4 °C for 5 min, and the supernatant was used for antioxidant assays. The absorbance was detected at 593 nm using UV–Vis spectrophotometer (Lambda 850, Storrs, CT, USA), and the total antioxidant capacity was calculated using standard curves.

### 4.3. Evans Blue Staining Analyses

We used 14-day-old hydroponically grown seedlings in these experiments. Six cultivars of seedlings with similar growth were treated for 0 h, 24 h, and 48 h. The roots of treated samples were washed with distilled water, and soaked in 0.025% *w*/*v* Evan’s blue dye for 15 min. The surface of stained samples was rinsed three times with distilled water, and then observed and photographed under a BX41 light microscope equipped with an Olympus DP80 CCD camera (Olympus, Tokyo, Japan). The roots of the six ryegrass cultivars were cut and weighed, then placed in a 10-mL centrifuge tube. Then, 3 mL 1% *w*/*v* sodium dodecyl sulfate aqueous solution was added, and the mixture was left for 3 days to allow extraction of the dye. The absorbance value of the extract at OD_600_ was measured using a SpectraMax Absorbance Reader (Molecular Devices, Sunnyvale, CA, USA).

### 4.4. Determination of Heavy Metal Concentrations in Seedlings’ Shoots and Roots

To explore the accumulation and translocation of Co^2+^, Pb^2+^, and Ni^2+^ in the shoots and roots of the six ryegrass cultivars, the heavy metal concentrations in tissues were determined by ICP-MS. After treatment for 24 h, ryegrass plants were soaked in 10 mM EDTA for 30 min to remove residual metals from the surface. The seedlings were separated into shoots and roots, and each part was dried at 80 °C for 3 days. The dried samples were weighed and then digested in 6 mL HNO_3_ (Aladdin, Shanghai, China) for 2 h, and then digested with a Milestone microwave digester (Socisole, Italy). The microwave heating procedure was as follows: increase to 120 °C at 5 °C/min, hold for 5 min, increase to 150 °C at 5 °C/min, hold for 10 min, increase to 190 °C at 5 °C /min, hold for 20 min. The mixture was cooled, the inner cover of the digestion tube was rinsed with a small amount of water, and then the microwave digestion tube was placed in a temperature-controlled porous acid extractor. The volume of the acid was reduced to the size of a bean at 160 °C, and then adjusted to 25 mL with pure water [42]. A series of standard solutions were prepared and tested, and 5% HNO_3_ was as blank control. The absorption tube was inserted into the solution after digestion. We selected the elements (Co, Pb, and Ni) to be analyzed. The Co, Pb, and Ni concentrations were detected by ICP-MS using a PerkinElmer instrument (PerkinElmer, Shelton, CT, USA).

### 4.5. Gene Expression Analyses in Lm1

We used 14-day-old hydroponically grown seedlings of Lm1 in this experiment. The seedlings were treated for 0 h, 24 h, and 48 h, and shoots and roots were collected for gene transcript analyses. Total RNA was extracted with Trizol reagent (Invitrogen, Carlsbad, CA, USA). The HiScript^®^ III 1 st Strand cDNA Synthesis Kit (Vazyme, Biotech Co., Ltd., Nanjing, China) was used to transcribe RNA into cDNA. The transcript levels of the following genes encoding heavy metal transporters were determined: *ATP-binding cassette* (*ABC*) *B-type transporter 4* (*ABCB4*), *ABCB21*, *ABC G-type transporter 37* (*ABCG37*), *ABCG48*, *cell number regulator 1* (*CNR1*), *CNR2*, *CNR13*, *natural resistance associated macrophage protein 4* (*NRAMP4*), *NRAMP6*, *Yellow stripe-like 6* (*YSL6*), *YSL12*, *metal tolerance protein 1* (*MTP1*), *heavy metal ATPase 5* (*HMA5*), *calmodulin-binding protein 60* (*CBP60*), and *multidrug resistance-associated protein* (*MRP*). The actin and gene-specific primers were provided in Table S5. The real-time PCR analyses were performed containing Hieff^TM^ qPCR SYBR Green Master Mix (YEASEN, Shanghai, China), and conducted using the CFX96 Touch Real-Time PCR assay system (Bio-Rad, Hercules, CA, USA). The relative gene transcript levels were calculated using the 2^−ΔΔCT^ method [49].

### 4.6. Statistical Analysis

Data shown in figures and tables are means ± standard error from three independent biological replicates. Mean values were compared among groups using *t*-test and one-way ANOVA, and the significance of difference was considered at *p* < 0.05. Statistical analyses were conducted using Excel 2010 and SPSS 13.0 software. Figures were constructed using Origin 8 software.

## Data Availability

Not applicable.

## Supplementary Materials

The following are available online at https://www.mdpi.com/article/10.3390/ijms222413583/s1.
