# Supplementary material for: Physiological and Gene Expression Responses of Six Annual Ryegrass Cultivars to Cobalt, Lead, and Nickel Stresses
Source: Int J Mol Sci. 2021 Dec 18;22(24):13583. doi: 10.3390/ijms222413583 (PMC8704220; doi:10.3390/ijms222413583)
Supplement: Supplementary file 1 [file ijms-22-13583-s001.zip › ijms-1502378-supplementary.pdf]

International Journal of *Molecular Sciences*

MDPI

Article Supporting Information

Physiological and gene expression responses of six annual ryegrass cultivars to cobalt, lead, and nickel stresses

Siyu Qiao[1, †], Ye Tao[1, †], Qinghua Shan[1], Jingang Wang[1], Tuanyao Chai[2], Shufang Gong[1, *], Kun Qiao[1, *]

[1] College of Horticulture and Landscape Architecture, Northeast Agricultural University, Harbin, 150030, PR China; 1359879583@qq.com (S.Q.); 314731728@qq.com (Y.T.); 1749287950@qq.com (Q.S.); wangjingang99@neau.edu.cn (J.W.); shufanggong@neau.edu.cn (S.G.); kunqiao@neau.edu.cn (K.Q.)

[2] College of Life Science, University of Chinese Academy of Sciences, Beijing, China; tychai@ucas.ac.cn (T.C.)

* Correspondence: shufanggong@neau.edu.cn (K.Q.); kunqiao@neau.edu.cn

† These authors have equally contributed to the work.

Citation: Lastname, F.; Lastname, F.; Lastname, F. Title. *Int. J. Mol. Sci.* **2021**, *22*, 13583. https://doi.org/10.3390/ijms222413583

Publisher's Note: MDPI stays neutral with regard to jurisdictional claims in published maps and institutional affiliations.

Copyright: © 2021 by the authors. Submitted for possible open access publication under the terms and conditions of the Creative Commons Attribution (CC BY) license (https://creativecommons.org/licenses/by/4.0/).

This supporting information contains six pages, five tables and six figures.

Tables: Table S1, Table S2, Table S3, Table S4 and Table S5

Figures: Figure S1, Figure S2, Figure S3, Figure S4, Figure S5, and Figure S6

Table S1. The number, cultivar's name, and provider of six ryegrass cultivars (*Lolium multiforum*)

Number	Cultivar's name	Provider
Lm1	Splendor	DLF-Trifolium Group
Lm2	Double Barrel	DLF-Trifolium Group
Lm3	Blue Heaven	Clover Group
Lm4	Barwoltra	Barenbrug
Lm5	Tetragold	Barenbrug
Lm6	Abundant	DLF-Trifolium Group

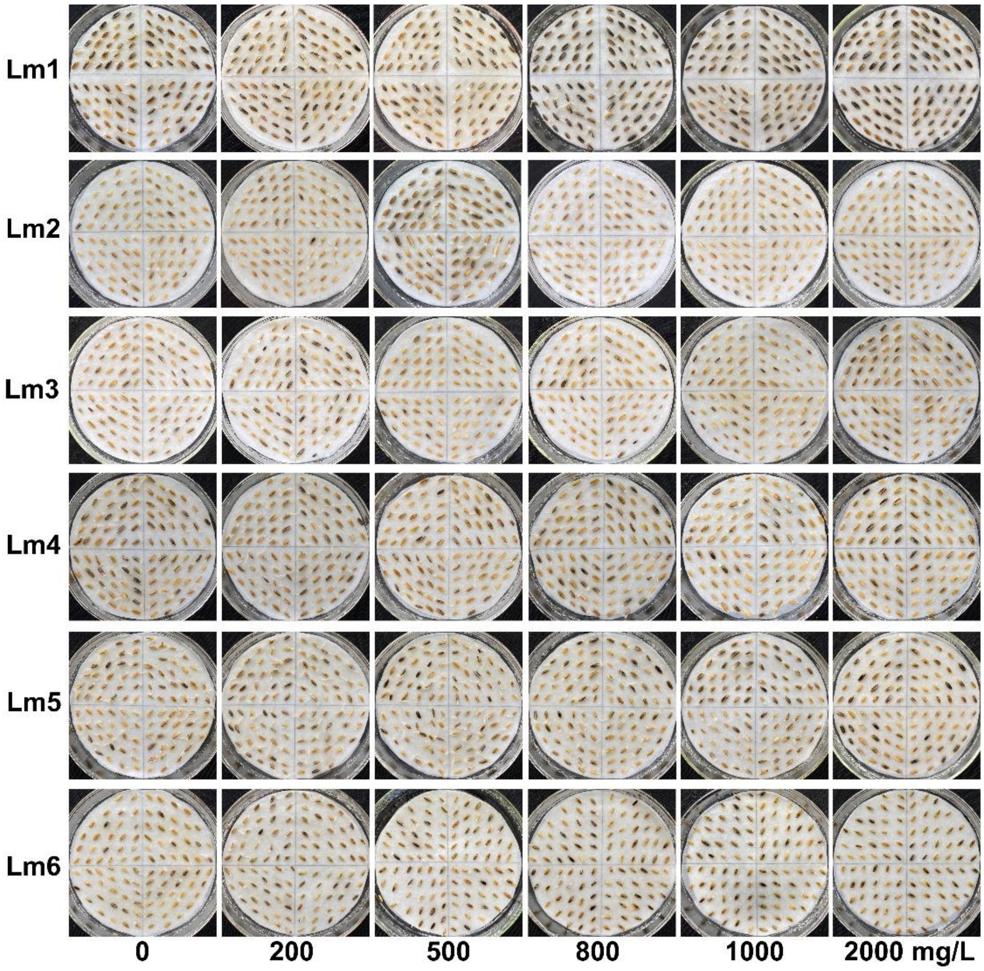

Figure S1. Seed germination of six ryegrass cultivars treated with different concentrations of Co^{2+}. Seeds were treated with deionized water containing 0, 200, 500, 800, 1000, or 2000 mg/L $CoCl_2$ and germination was monitored over 5 days. Each plate was divided into four zones, each of which contained 100 seeds.

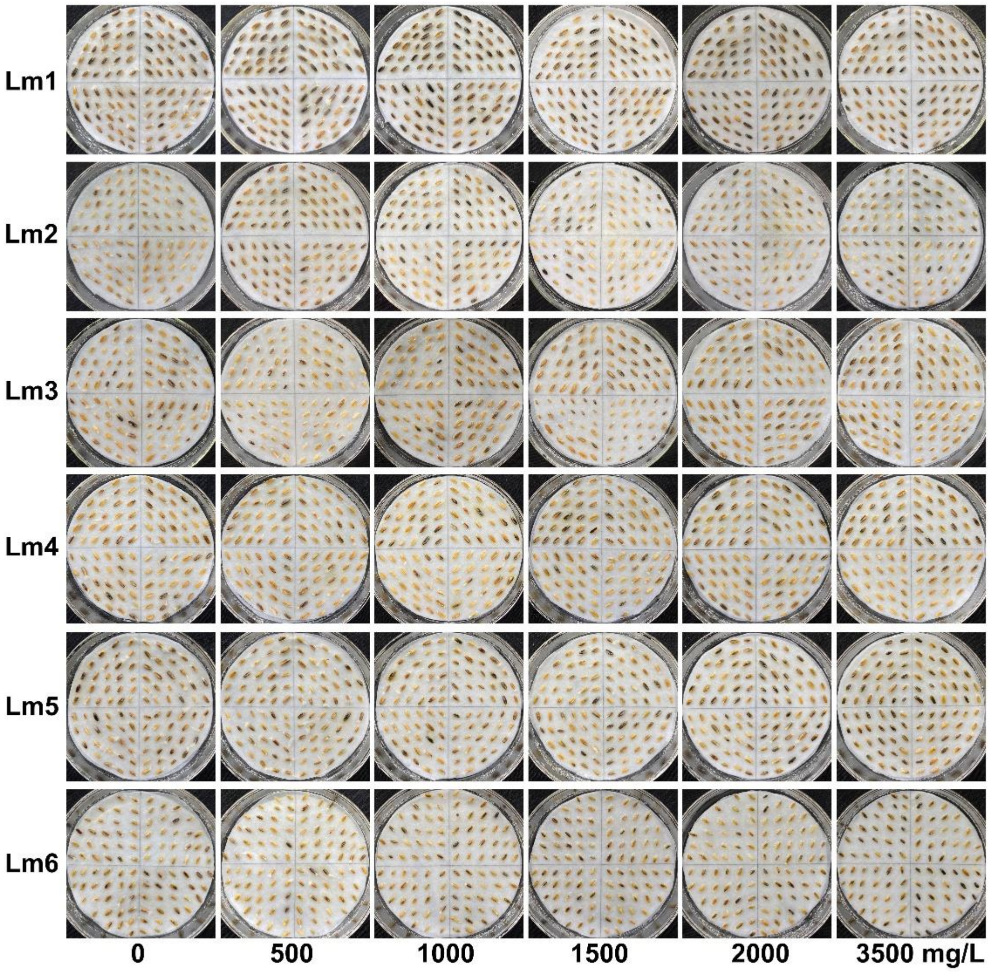

Figure S2. Seed germination of six ryegrass cultivars treated with different concentrations of Pb^{2+}. Seeds were treated with deionized water containing $Pb(NO_3)_2$ at 0, 500, 1000, 1500, 2000, 3500 mg/L, and germination was monitored for 5 days.

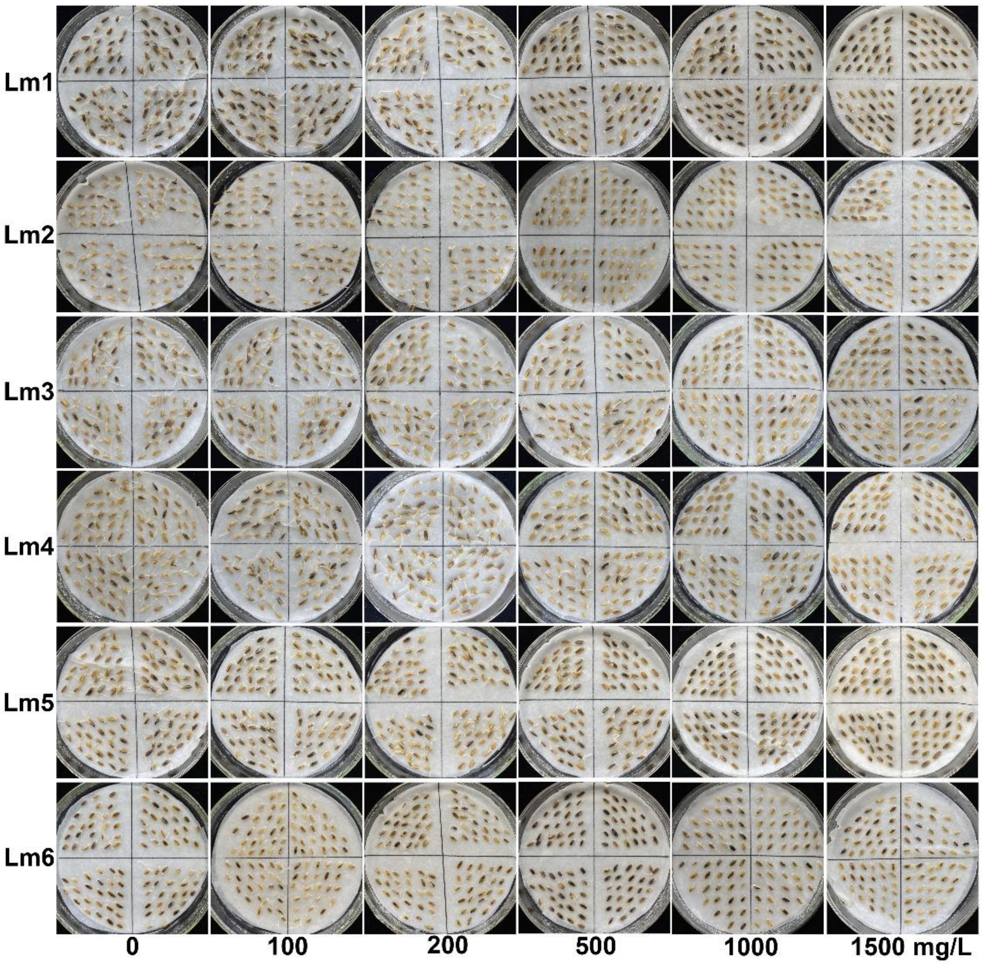

Figure S3. Seed germination of six ryegrass cultivars in treated with different concentrations of Ni^{2+}. Seeds were treated with deionized water containing $NiSO_4$ at 0, 100, 200, 500, 1000, and 1500 mg/L, and germination was monitored for 5 days.

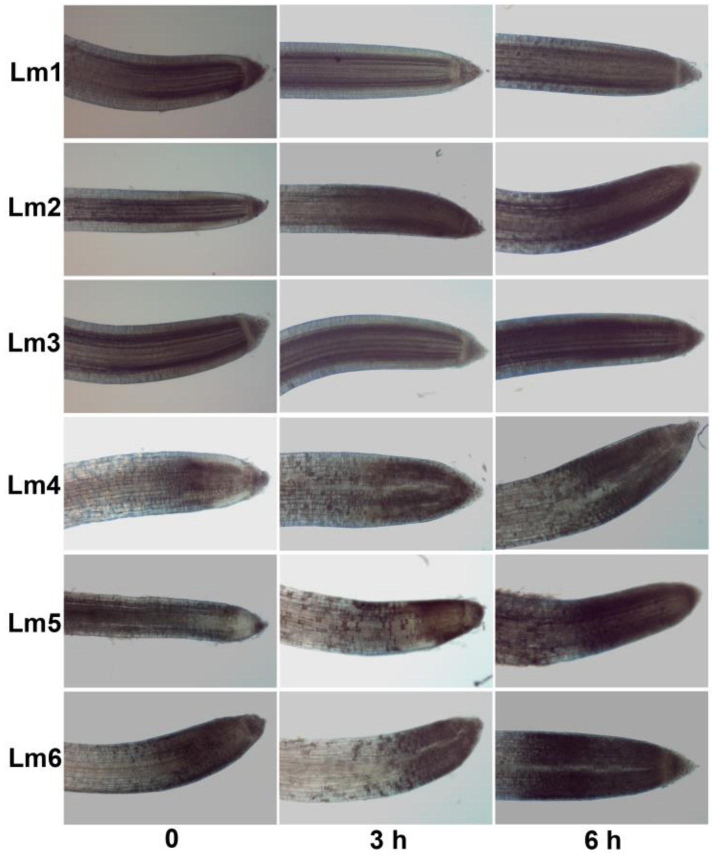

Figure S4. Evans blue staining of six ryegrass cultivars' roots under $CoCl_2$ stress. These seedling roots are treated with 500 mg/L $CoCl_2$ for 3 and 6 h. The cellular structure of roots is observed using BX41 optical microscope with Olympus DP80 CCD camera.

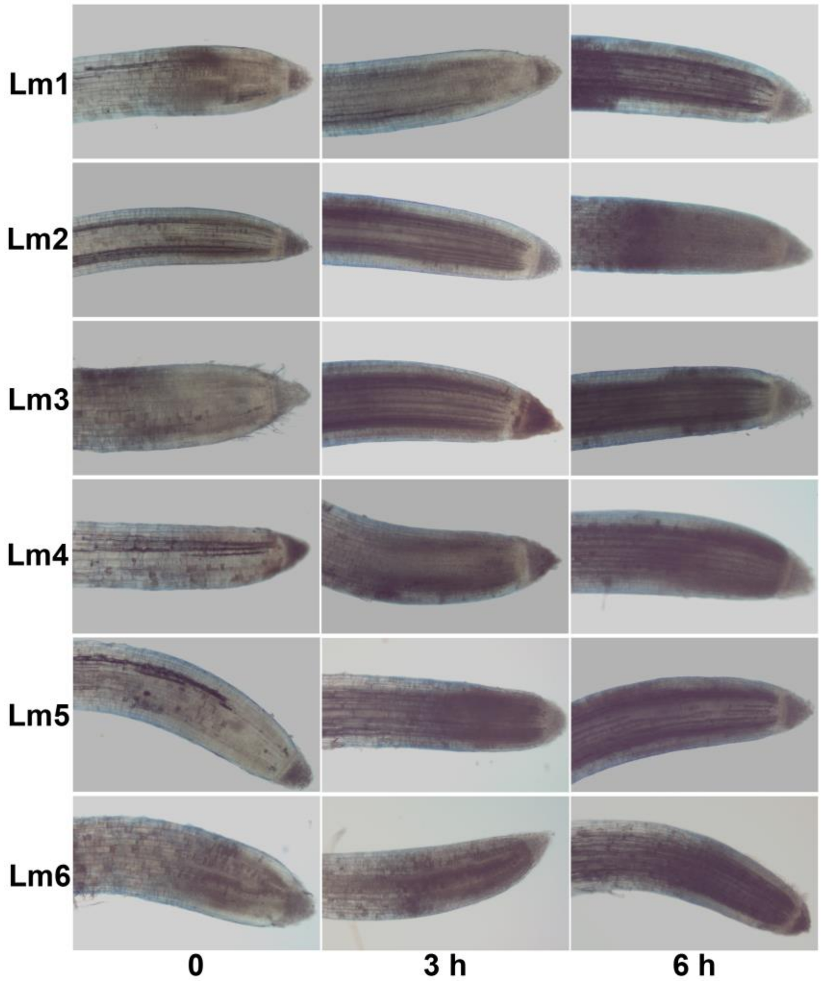

Figure S5. Evans blue staining of six ryegrass cultivars' roots under $Pb(NO_3)_2$ stress. These seedling roots are treated with 1000 mg/L $Pb(NO_3)_2$ for 3 and 6 h. The cellular structure of roots is observed using BX41 optical microscope with Olympus DP80 CCD camera.

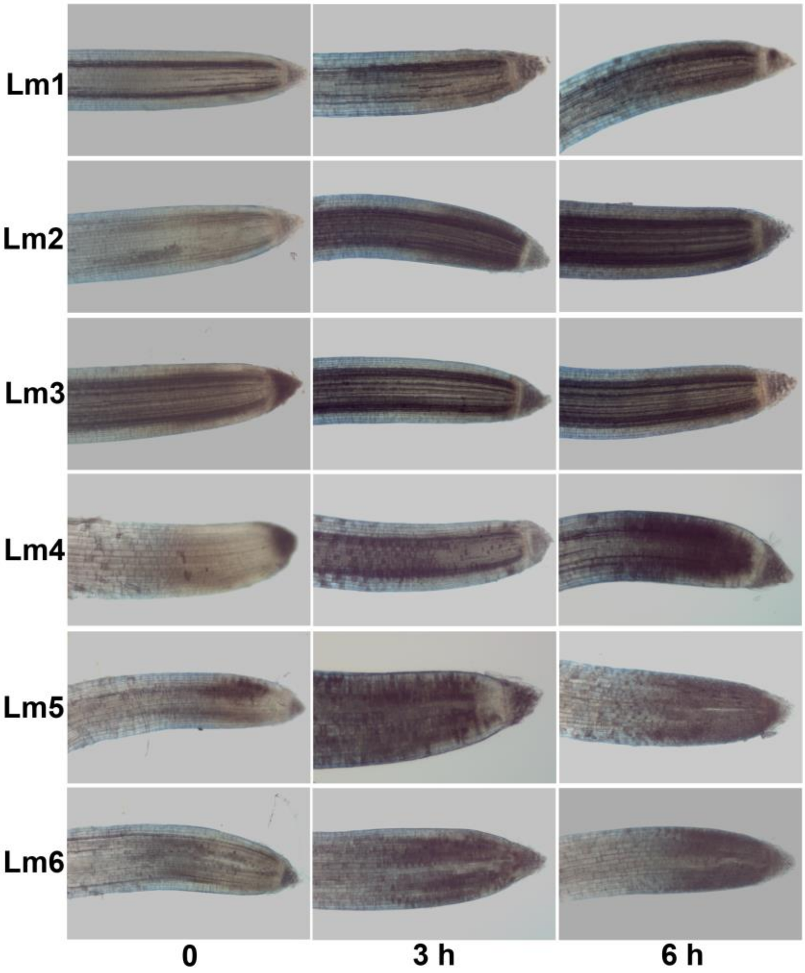

Figure S6. Evans blue staining of six ryegrass cultivars' roots under $NiSO_4$ stress. These seedling roots are treated with 500 mg/L $NiSO_4$ for 3 and 6 h. The cellular structure of roots is observed using BX41 optical microscope with Olympus DP80 CCD camera.

Table S2. Comprehensive evaluation of Co tolerance of six ryegrass cultivars

	Comprehensive evaluation							
Number	Relative germination rate	Relative fresh weight	Relative plant height	Relative root length	T-AOC	Extraction	Mean membership index value	Ranking
Lm1	0.6	0.502	0.696	0.390	0.322	0.966	0.579	1
Lm2	0.626	0.424	0.390	0.266	0	0.893	0.433	6
Lm3	0.491	0.361	0.496	0.348	0.266	0.95	0.485	4
Lm4	0.668	0.432	0.579	0.371	0.259	1	0.551	3
Lm5	0.738	0.542	0.521	0.337	0.548	0	0.448	5
Lm6	0.571	0.426	0.385	0.301	1	0.652	0.556	2

Table S3. Comprehensive evaluation of Pb tolerance of six ryegrass cultivars

	Comprehensive evaluation							
Number	Relative germination rate	Relative fresh weight	Relative plant height	Relative root length	T-AOC	Extraction	Mean membership index value	Ranking
Lm1	0.616	0.339	0.491	0.280	0.886	1	0.602	1
Lm2	0.445	0.379	0.401	0.244	0.580	0.496	0.424	3
Lm3	0.498	0.357	0.412	0.258	0	0.775	0.383	6
Lm4	0.455	0.355	0.394	0.250	0.346	0.552	0.392	5
Lm5	0.501	0.396	0.465	0.295	0.751	0.388	0.466	2
Lm6	0.417	0.339	0.391	0.246	1	0	0.399	4

Table S4. Comprehensive evaluation of Ni tolerance of six ryegrass cultivars

	Comprehensive evaluation							
Number	Relative germination rate	Relative fresh weight	Relative plant height	Relative root length	T-AOC	Extraction	Mean membership index value	Ranking
Lm1	0.572	0.481	0.492	0.395	0.509	0.820	0.545	1
Lm2	0.428	0.360	0.402	0.327	0.750	0.726	0.499	4
Lm3	0.584	0.514	0.546	0.432	0	1	0.513	2
Lm4	0.474	0.379	0.459	0.359	0.730	0.664	0.511	3
Lm5	0.524	0.558	0.479	0.418	1	0	0.497	5
Lm6	0.402	0.562	0.400	0.351	0.655	0.146	0.419	6

Table S5. Primers used for the real-time quantitative PCR. F: Forward primer. R: Reverse primer.

Gene ID	Gene Symbol	Primers
Unigene26285_All	probable metal-nicotianamine transporter YSL6	F:GTATCTGTCATACTCGGTGATGG<br>R:CCATCCTGAACCCTGACAAGAG
CL3029.Contig8_All	ABC transporter B family member 4-like	F: CGGGTTTCAGTCAAGATGCTAA<br>R: CCAATGCCTCCAACTATTCCTG
CL3314.Contig7_All	ABC transporter B family member 21	F: TCGCTGTCATTCATCAAGGTTC<br>R: TCTGTCTTATCCTGACGGCTTG
CL55.Contig3_All	ABC transporter G family member 48-like	F: AAGAAGCGTGTCACAACTGGG<br>R: CGGTGGTTGTAGGAGGGAGAT
CL9563	Copper-transporting ATPase HMA5	F: ATTCGTCTCGGTGAGCAGCA<br>R: CCCCTCGGGATAATCGTAGA
CL4302.Contig10_All	CNR1	F: TGACATATCGGGACAGCGCG<br>R: TTGGGAGGCAAACATGGAGC
CL12636.Contig3_All	CNR2	F: CATCGATTGCTTCGACGACCG<br>R: CAAGATCCGAGATTGGCTCTCC
CL7906.Contig2_All	CNR13	F: CACGGAGTTCTACACCTGTTCC<br>R:<br>AATCCTTGAGAAGGTTCCACAGG
CL13487.Contig2_All	Metal transporter NRAMP1	F: CTAGGTCCTTGGCAATTGTGC<br>R:<br>AGAACCGATCACCCATGTTAGC
CL10094.Contig3_All	Metal transporter NRAMP6	F: AATCCAGCGCGATTGTCTACG<br>R: GAACCCCTGCATGATGTACTGG
Unigene810_All	Metal tolerance protein 2-like	F: AGAAGGAAGGGAGTGGGCTG<br>R: GCCTGAGACAACAAGCCCAGC
CL15025C1_All	Actin	F: TACGACCAGGAGATGGAGACC<br>R: GAAGGAAGGCTGGAAGAGGAC
